# Cell-Free Systems Based on CHO Cell Lysates: Optimization Strategies, Synthesis of “Difficult-to-Express” Proteins and Future Perspectives

**DOI:** 10.1371/journal.pone.0163670

**Published:** 2016-09-29

**Authors:** Lena Thoring, Doreen A. Wüstenhagen, Maria Borowiak, Marlitt Stech, Andrei Sonnabend, Stefan Kubick

**Affiliations:** 1 Department of Cell-free and Cell-based Bioproduction, Branch Bioanalysis and Bioprocesses, Fraunhofer-Institute for Cell Therapy and Immunology (IZI-BB), Potsdam-Golm, Germany; 2 Institute for Biotechnology, Technical University of Berlin (TUB), Gustav-Meyer-Allee 25, 13355, Berlin; Berlin Institute of Technology, GERMANY

## Abstract

Nowadays, biotechnological processes play a pivotal role in target protein production. In this context, Chinese Hamster Ovary (CHO) cells are one of the most prominent cell lines for the expression of recombinant proteins and revealed as a safe host for nearly 40 years. Nevertheless, the major bottleneck of common *in vivo* protein expression platforms becomes obvious when looking at the production of so called “difficult-to-express” proteins. This class of proteins comprises in particular several ion channels and multipass membrane proteins as well as cytotoxic proteins. To enhance the production of “difficult-to-express” proteins, alternative technologies were developed, mainly based on translationally active cell lysates. These so called “cell-free” protein synthesis systems enable an efficient production of different classes of proteins. Eukaryotic cell-free systems harboring endogenous microsomal structures for the synthesis of functional membrane proteins and posttranslationally modified proteins are of particular interest for future applications. Therefore, we present current developments in cell-free protein synthesis based on translationally active CHO cell extracts, underlining the high potential of this platform. We present novel results highlighting the optimization of protein yields, the synthesis of various “difficult-to-express” proteins and the cotranslational incorporation of non-standard amino acids, which was exemplarily demonstrated by residue specific labeling of the glycoprotein Erythropoietin and the multimeric membrane protein KCSA.

## Introduction

Nowadays, production of recombinant proteins plays a pivotal role in the pharmaceutical industry. In particular, genetically engineered mammalian cells have become the predominant system for the manufacturing of proteins for clinical applications [1]. Human tissue plasminogen activator was one of the first therapeutic proteins, produced in mammalian cell culture by Genentech in 1986 [2,3]. Currently Chinese Hamster Ovary (CHO) cells are the most popular and standardized cell line for recombinant protein production [4,5]. Nearly 70% of all pharmaceuticals are produced in engineered CHO cells [6,7]. There are several reasons for the decision to use CHO cells as an industrial working horse. For large scale industrial production of recombinant drugs, fermentation processes are preferentially performed in suspension cultures. CHO cells can be easily adapted and grown in suspension cultures, while serum-free and chemically defined media can be applied, which is advantageous with regard to Batch-to-Batch reproducibility [2]. Additonally, fermentation of CHO cells is cost-saving and favorable due to safety reasons [8]. Moreover CHO cells are safety approved for nearly 30 years (FDA) and therefore suitable for industrial large scale production processes [5]. CHO cells were chosen as a preferable system for protein production due to the fact that this expression platform enables a high similarity of biochemical properties compared to the origin of recombinant target proteins [9]. Consistently, recombinant proteins, produced in CHO cells, often show correctly folded structures and appropriate posttranslational modifications, e.g. harboring human like glycosylations [10]. These qualities emphasize the ability to generate functional, glycoprotein therapeutics [2,11].

The production of certain specific target proteins of eukaryotic origin often posed issues in *in vivo* expression platforms. These so called “difficult-to-express” proteins cover various types of membrane proteins, including ion channels, multi-pass membrane proteins and G protein coupled receptors, as well as proteins that exhibit cytotoxic effects on the host cell during overexpression. To circumvent the bottlenecks of *in vivo* protein production systems, tailor-made cell-free protein synthesis systems have been developed, based on different translationally active cell extracts of prokaryotic and eukaryotic origin [12–14]. The choice of the individual cell-free platform depends on the requirements necessary for the target protein and its subsequent application. In general, prokaryotic cell-free systems are mainly based on lysates generated from *Escherichia coli* (*E*.*coli*) [15–19]. These systems, suitable for the high yield production of proteins, are limited in the implementation of posttranslational modifications [20]. Eukaryotic cell-free systems represent an alternative option for the production of difficult-to-express proteins. In general, these systems can be divided into two classes: Cell-free systems harboring endogenous microsomal structures and systems without any endogenous membrane compartments [21,22]. Prominent candidates of lysates without endogenous vesicles are rabbit reticulocyte lysates and wheat germ lysates [23–28].

Lysates, based on rabbit reticulocytes, are long standing and well-known cell-free systems [29,30] which were often used for fundamental research studies [31–33]. Nevertheless, rabbit reticulocyte lysate system exhibits many disadvantages including a laborious and cost intensive preparation strategy, low protein synthesis rates and the requirement to supplement exogenous vesicular structures, e.g. canine microsomes [34], in order to obtain functional posttranslationally modified proteins.

Wheat germ systems can be used for high yield production of proteins and automation technologies are available [35]. The major bottleneck of the wheat germ system is the limitation in posttranslational modifications due to the absence of endogenous microsomal structures. Therefore, the generation of novel cell-free systems needs to combine the preparation of endogenous microsomes for the synthesis of posttranslationally modified proteins and the high-yield production of functional proteins. In this context, various studies revealed multiple systems originating from different eukaryotic sources, including insect cells [36–40], plant cells [41–44] and mammalian cells [45,46]. Recent studies performed by Broedel et. al. [47,48] showed initial results regarding the cell-free synthesis based on CHO cells. To close the gap between applied research and industrial protein production, this system has subsequently been further developed.

We describe the diversity of cell-free protein synthesis based on CHO cell lysate. Optimization and improvement of protein yields as well as the ability to produce different classes of “difficult-to-express” proteins is demonstrated. Subsequently the cotranslational incorporation of non-standard amino acids in CHO lysate systems was exemplified by the cell-free production of fluorescently labeled glycoproteins and membrane proteins.

## Materials and Methods

### Template generation

Cell-free protein syntheses in CHO lysates were performed using either vector DNA or linear DNA templates generated by polymerase chain reaction (PCR). Different vector backbones harboring a firefly luciferase (Luc) gene and internal ribosomal entry sites were tested in cell-free systems. This study included the following plasmids: pIX3.0-CRPV(GCT)-Luc, pIX3.0-EMCV-Luc, pcDNA3.1-CRPV(GCT)-Luc and pT7CEF1-EMCV-Luc. If not stated differently all vector based DNA constructs are based on a common *in vitro* protein synthesis pIX3.0 vector backbone (Biotech Rabbit) containing regulatory sequences for transcription and translation. A T7 promotor and a T7 terminator constituted regulatory sequences for transcription. For translation initiation an internal ribosomal entry site (IRES) of cricket paralysis virus was included into the 5´UTR and the start codon triplet of the gene of interest was exchanged from ATG to GCT, as it was shown in previous studies (Broedel et. al. 2014). The following proteins were selected as model proteins for initial tests: Luciferase (Luc) (cytosolic protein, 60.6 kDa), enhanced yellow fluorescent protein eYFP (cytosolic protein, 26.9 kDa) and erythropoietin including melittin signal peptide (Mel-EPO) for efficient translocation of proteins into microsomal structures of the CHO lysates (glycosylated protein, 19.7 kDa) with two deleted N-glycosylation sites at asparagine 52 and glutamine 110. To evaluate the performance of the CHO lysate cell-free protein synthesis system, different classes of membrane proteins were synthesized covering ion channels, G protein coupled receptors as well as multi-pass transmembrane proteins. During initial experiments different vector backbones and IRES were evaluated regarding their performance in CHO lysate based cell-free systems.

Templates for analyzing the ability of the CHO based cell-free system to synthesize proteins from linear DNA constructs were amplified by PCR. PCR was performed by using Hot Star HiFidelity polymerase (Qiagen) and following the manufacturer´s protocol. For this purpose the plasmids pIX3.0-CRPV(GCT)-Luc and pIX3.0-CRPV(GCT)-EPO were used in the PCR as general DNA templates. Adapter primers N-0 (5´ATG ATA TCT CGA GCG GCC GCT AGC TAA TAC GAC TCA CTA TAG GGA GAC CAC AAC GGT TTC CCT CTA GAA ATA ATT TTG TTT AAC TTT AAG AAG GAG ATA AAC AAT G 3´) and C-0 (TAA TAA CTA ACT AAC CAA GAT CTG TAC CCC TTG GGG CCT CTA AAC GGG TCT TGA GGG GTT TTT TGG ATC CGA ATT CAC CGG TGA TAT CAT) were used to amplify linear DNA-templates. Each of the adapter primers has overlap sequences which fuse regulatory sequences necessary for translation and transcription to the gene of interest.

### Cell-free protein synthesis

Cell-free synthesis reactions were performed using CHO cell derived translationally active lysates. Lysates were prepared from cultured, exponentially grown CHO cells as described in previous studies [47]. The protein synthesis processes were conducted in coupled transcription/translation reactions in a final volume of 20 to 50 μl. The reactions were incubated in a thermomixer (Eppendorf) for 3 h at 30°C and 600 RPM. Protein synthesis reactions were composed of 40% (v/v) of translationally active lysate supplemented with HEPES-KOH (pH 7.5, f.c. 30 mM), sodium acetate (f.c. 100 mM), Mg(OAc)_2_ (f.c. 3.9 mM), KOAc (f.c. 150 mM), amino acids (100 μM), spermidin (f.c. 0.25 mM), DTT (2.5 mM) and energy generation components including creatine phosphokinase (f.c. 0.1 mg/ml), creatine phosphate (20 mM), ATP (1.75 mM) and GTP (0.3 mM). If not reported otherwise, 60 ng/μl of plasmid DNA was added to the translation reaction. To enable DNA transcription to mRNA during cell-free protein synthesis, 1 U/μl T7 RNA polymerase, 0.3 mM of UTP and CTP and 0.1 mM of the cap analogue m7G(ppp)G were added to the reaction. Cell-free protein synthesis reactions were supplemented with radioactive ^14^C leucine (f.c. 50 μM, specific radioactivity 66.67 dpm/pmol) for radiolabeling of produced proteins, in order to allow the further analysis of synthesized proteins by autoradiography and liquid scintillation counting.

Enrichment of membrane and secreted proteins into microsomal structures was investigated by repetitive translation cycles. After each translation step translation mixture was separated into microsomal fraction and supernatant by centrifugation (16000xg, 15 min, 4°C). A 5 μl aliquot of each fraction was taken for further analysis. Microsomal fraction was newly resuspended using freshly prepared translation mixture without vesicles to start a new translation cycle and thereby enabling multiple addressing of microsomes.

### Luciferase assay and western blot

Functionality of cell-free synthesized firefly luciferase was analyzed using Luciferase Assay Reagent (LAR, Promega). 50 μl of LAR were added to 5 μl of translation mixture. Subsequently luciferase induces a light reaction by catabolizing LAR, which was detected by a luminometer (LB941, Berthold, Germany). The concentration of active protein was determined by using a calibration curve. Additionally, cell-free produced luciferase was monitored by western blot analysis according to the manufacturer´s instructions using an IBlot device (Lifetechnologies). For this purpose, a primary rabbit anti luciferase IgG monoclonal antibody (St. John´s Laboratory) was applied for analysis. Detection of luciferase protein band was accomplished by using a secondary anti rabbit IgG antibody coupled to horseradish peroxidase (HRP). The addition of ECL reagent (Promega) led to a light reaction that was detected by a Typhoon TRIO+ Imager system (GE Healthcare).

### Analysis of radio labeled proteins

The yields of radiolabeled proteins were determined by hot TCA precipitation. Therefore 5 μl of translation mixtures were transferred to a glass tube, 3 ml of 10% TCA/ 2% casein hydrolysate solution were added and boiled for 15 min at 80°C followed by incubation in an ice bath for 30 min. Precipitated proteins were transferred to membrane filters (Macherey Nagel), using a vacuum filtration device (Hoefer). Dried filters were placed into scinitillation tubes and 3 ml of scintillation cocktail (Zinsser Analytik) was added to the tubes. Radioactivity of samples was analyzed using a LS6500 Multi-Purpose scintillation counter (Beckman Coulter). The molecular mass of radiolabeled, cell-free synthesized proteins was determined by SDS-PAGE followed by autoradiography. The first step of sample preparation included an acetone precipitation of synthesized proteins. Therefore 5 μl of translation mixture and 45 μl of Millipore water were supplemented with 150 μl ice cold acetone. Samples were chilled on ice for at least 15 min. Afterwards precipitated proteins were centrifuged at 16000xg and 4°C for 10 min. Acetone was removed from the samples and pellets were dried at 45°C for 1 h in a thermomixer. Dried protein pellets were resuspended in LDS sample buffer (NUPAGE LDS sample buffer supplemented with 50 mM DTT) and samples were separated on precast non-reducing NUPAGE SDS-PAGE gels for 35 min at 200 V. SDS-PAGE gels harboring the separated proteins were stained with Coomassie blue solution (SimplyBlue SafeStain, Life technologies). Subsequently, gels were dried on a Whatman paper for 60 min at 70°C (Unigeldryer 3545D, Uniequip). Dried gels, harboring radiolabeled proteins, were exposed to phosphor screens. Finally, radiolabeled proteins were visualized using a phosphor imager system (Typhoon TRIO+ Imager, GE Healthcare).

### Fluorescence analysis of eYFP fusion proteins

Cell-free synthesized eYFP and eYFP fusion proteins were analyzed using a Typhoon TRIO + Imager system (Excitation 488 nm, emission filter 526 nm short-pass; GE Healthcare). 5 μl of translation mixture was diluted with 20 μl of PBS and transferred to a μ-Ibidi slide for further analysis (Ibidi GmbH, Munich, Germany). The quantification of fluorescence intensity was performed by using Image Quant TL software.

### Residue specific labeling of proteins using BODIPY-TMR-Lysine

An alternative labeling of cell-free produced proteins was conducted by cotranslational incorporation of BODIPY-TMR-Lys. A final concentration of 2 μM BODIPY-TMR-Lys was supplemented to the cell-free synthesis reaction. BODIPY-TMR-Lys labeled proteins were separated on SDS-PAGE and analyzed by detecting in-gel fluorescence. SDS-PAGE was performed as described in part 2.4 followed by a 30 min incubation in a 50% methanol/water solution at room temperature. Fluorescence signals of labeled proteins were visualized using a Typhoon TRIO + imager (GE Healthcare).

## Results

### Application of different plasmid types for cell-free protein synthesis in translationally active CHO cell lysates

**Fig 1:**
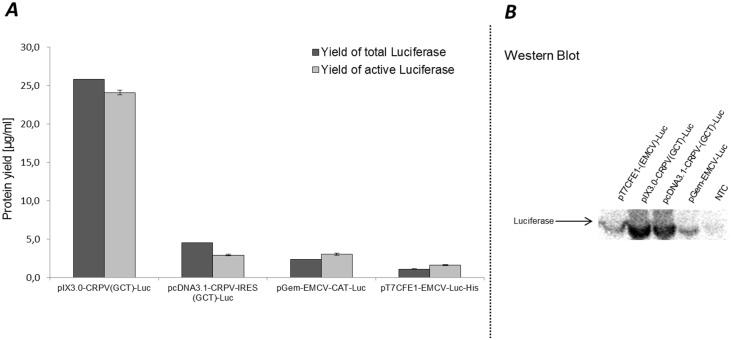
Quantitative analysis of cell-free protein synthesis: A comparison of different luciferase encoding vector backbones probed in CHO cell lysates. **A.** Total protein yields were determined by incorporation of radioactive ^14^C leucine into *de novo* synthesized proteins followed by hot TCA precipitation and scintillation measurement. Yields of active luciferase were quantified by standard luciferase assay. **B.** Western blot analysis of cell-free produced luciferase using primary Anti-Luc antibody (concentration 1:1000) and secondary Anti-rabbit-HRP conjugate antibody (1:2000). Analysis of luciferase bands was performed by using ECL reagent and detection of corresponding light emission. Error bars show standard deviations calculated from triplicate analysis. NTC sample contains translation mixture without synthesized protein.

Cell-free production of proteins generally requires a DNA template or RNA to start synthesis of the desired target protein. Nowadays, different types of plasmids are available that can be applied directly to cell-free systems of prokaryotic and eukaryotic origin. A few DNA structure characteristics need to be fulfilled to enable the synthesis of chosen proteins in cell-free systems based on extracts from CHO cells. These requirements can be divided into two parts, which cover DNA elements essential for transcription and translation. Our newly developed cell-free system based on CHO cell lysates requires a T7- promoter- and terminator sequence introduced upstream of the gene sequence to realize transcription of RNA conducted by T7 RNA polymerase. DNA templates for the translation reaction comprises a ribosomal binding site for initiation of the translational process. As described earlier, an insertion of the Internal Ribosomal Binding Sites (IRES) for cap independent initiation of the translation reaction is advantageous for high yield production of proteins in different eukaryotic cell-free systems [48]. One of the standardized vectors that can be used for CHO cell-free systems is the *in vitro* expression vector EasyXpress pIX3.0 (Biotechrabbit GmbH, Germany). The additional insertion of the intergenic region (IGR) IRES site of cricket paralysis virus (CRPV) into the pIX3.0 vector leads to high levels of cell-free produced protein in coupled batch based systems [48]. To exploit the flexibility of our newly developed cell-free system, different plasmids, harboring CRPV and EMCV IRES site and a gene encoding firefly luciferase protein, were applied to translation reactions based on extracts from CHO cells. The first part of this study included cell-free synthesis based on different vector backbones such as pIX3.0-CRPV(GCT)-Luc, pcDNA3.1-CRPV(GCT)-Luc, pGem-EMCV-Luc and pT7CFE1-EMCV-Luc (Table 1). pIX3.0 and pT7CFE1 based vectors are currently used for cell-free synthesis in commercially available cell-free protein synthesis kits (Biotech Rabbit GmbH, Thermoscientific). Highest luciferase protein yields were detected by using an optimized pIX3.0-CRPV(GCT)-Luc template for cell-free protein synthesis (25.8 μg/ml radio labeled protein and 24.0 μg/ml active protein). Using other vector backbones, protein yields of approximately 1.1 to 4.5 μg/ml of radio labeled and 1.5 to 3 μg/ml of active protein yield could be reached (Fig 1A, Table 1). A direct comparison of IRES dependent protein production was performed using plasmids harboring the CRPV-IGR-IRES and EMCV-IRES. Our results show that higher luciferase yields could be obtained by using the CRPV-IGR-IRES site. We additionally examined the molecular weight of *de novo* synthesized luciferase proteins using standard western blot analysis. The results of the analysis are shown in Fig 1B. Luciferase protein bands could be detected in all of the different samples, whereas in the negativ control, which represents translation mixture without DNA template for monitoring the detection background of the synthesis reaction, no luciferase protein band could be detected. The intensity of protein bands differs, while samples including pIX3.0-CRPV(GCT)-Luc and pcDNA3.1-CRPV(GCT)-Luc DNA templates show the highest intensity.

**Table 1:** Protein yields using different luciferase encoding plasmids in cell-free synthesis based on CHO cell lysates.

Plasmid	Yield of total luciferase [μg/ml]	Yield of active luciferase [μg/ml]
pIX3.0-CRPV(GCT)-Luc	25.8	24.0
pcDNA3.1-CRPV(GCT)-Luc	4.5	2.9
pGem-EMCV-Luc	2.3	3.0
pT7CFE1-EMCV-Luc	1.1	1.6

### Optimization of cell-free reaction conditions

**Fig 2:**
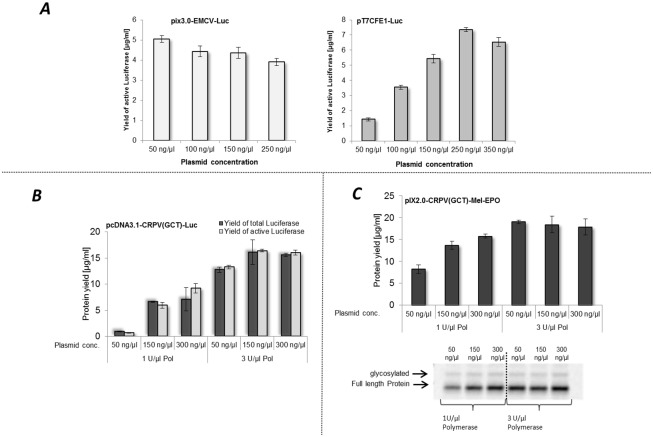
Evaluation of plasmid and T7 RNA polymerase concentration applied for CHO lysate based cell-free synthesis. **A.** Analysis of plasmid concentration of pIX3.0-EMCV-Luc and pT7CFE1-Luc used for cell-free protein synthesis. Protein yields of active luciferase were determined by standard luciferase assay **B.** Dependence of protein yield on plasmid and T7 RNA polymerase concentration during cell-free synthesis based on the template pcDNA3.1-CRPV(GCT)-Luc. Protein yields of *de novo* synthesized luciferase were detected and calculated by quantification of ^14^C leucine labeled proteins and scintillation measurement. Luciferase assay was used for investigation of the amount of functional protein. Error bars represent standard deviations calculated from triplicate analysis. **C** Investigation of protein yield using pIX2.0-CRPV(GCT)-Mel-EPO DNA template. Variation of template DNA and T7 RNA polymerase concentration. Radio labeled proteins were analyzed by TCA precipitation followed by scintillation measurement (upper part C). Additionally, *de novo* synthesized proteins were visualized by autoradiography (lower part C).

We performed a set of experiments to increase the total yield of cell-free synthesized luciferase using different DNA templates as described in part 3.1. Three individual experimental approaches were performed based on DNA template alterations. Initially, we have investigated the influence of various plasmid-DNA template concentrations in cell-free protein synthesis reactions for different templates (Fig 2A). The optimal DNA-concentration of pIX3.0-CRPV(GCT)-Luc used for *in vitro* translation in CHO cell lysates was determined to be approximately 50 ng/μl as also shown by Broedel et. al. [48]. Different concentrations of DNA-templates were studied to examine the optimal value for each individual vector. In these initial experiments, we detected a decrease of active luciferase yield from 5 μg/ml to 3.9 μg/ml by increasing plasmid concentration of pIX3.0-EMCV-Luc (Fig 2A). However, by increasing the DNA concentrations of pT7CFE1-Luc in cell-free protein synthesis reactions from 50 ng/μl to 350 ng/μl, an increase of active protein concentration was detected, reaching the maximum protein yield at 250 ng/μl of DNA template. Using this DNA template concentration, protein yields of 7 μg/ml were reached which is 7 fold higher compared to start conditions. Further investigations included optimization of protein yield using a pcDNA3.1-CRPV(GCT)-Luc DNA template (Fig 2B). This plasmid is universally applicable as it can be used for cell-free protein synthesis as well as for cell-based protein production. The experimental setup was further expanded by analyzing different T7 RNA polymerase concentrations in coupled transcription/translation reactions. Different concentrations of T7 RNA polymerase were tested for pIX3.0-CRPV(GCT)-Luc DNA template. A saturation point of luciferase yield was obtained using 3 to 4 U/μl T7 RNA polymerase (S1 Fig). Therefore concentrations of 1 U/μl and 3 U/μl T7 RNA polymerase were tested for the different DNA templates. T7 RNA polymerase is a DNA dependent RNA polymerase which is commonly used in cell-free protein synthesis systems [49]. So far, standard cell-free reactions based on CHO cell lysate, were conducted using 1 U/μl T7 RNA polymerase [46]. In contrast, our results showed, that increased protein yields were reached by using 3 U/μl T7 polymerase and 150 ng/μl of pcDNA3.1-CRPV(GCT)-Luc DNA template. Comparison of the results of the two different tested T7 RNA polymerase concentrations showed significant improvement of protein yield using an increased concentration of 3 U/μl T7 RNA polymerase for cell-free protein synthesis (Fig 2B). This finding is also supported by the results depicted in Fig 2C. Efficient cell-free synthesis of erythropoietin (EPO) containing a melittin signal peptide is strongly dependent on T7 RNA polymerase concentration and plasmid DNA concentration. Cell-free synthesis was analyzed by synthesizing Mel-EPO. The highest protein yield was obtained by using 3 U/μl T7 polymerase and 50 ng/μl plasmid DNA. In the case of Mel-EPO, a slight decrease of protein yield could be detected by increasing plasmid concentration and T7 RNA polymerase. The supplementation of coupled transcription/ translation reactions with 3 U/μl T7 RNA polymerase (Stratagene) proved to be an efficient way to increase protein yields in CHO cell-free systems.

**Fig 3:**
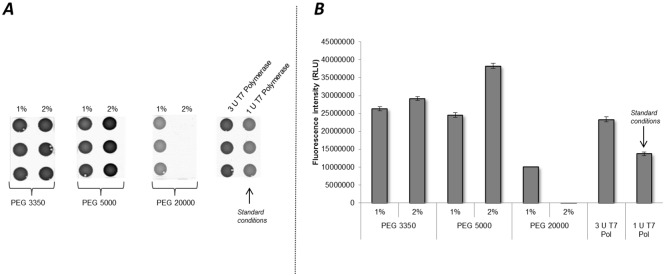
Influence of PEG on protein production in cell-free systems based on translationally active CHO lysate. Two concentrations (1%, 2%) of different PEG molecules (3350, 5000, 20000) were analyzed in cell-free protein synthesis reactions using pIX3.0-CRPV(GCT)-eYFP plasmid template. Translation reactions without the addition of PEG but with supplementation of 3 U/μl (increased concentration) and 1 U/μl (standard concentration) T7 RNA polymerase served as control reactions ***A***. Fluorescence signals of synthesized eYFP proteins were detected by fluorescence imaging on μ-Ibidi slides using the Typhoon Trio Plus Imager. ***B***. Quantification of fluorescence signals was accomplished by using Image Quant TL Array analysis software. Error bars show standard deviations calculated from triplicate analysis.

To further analyze the effect of T7 RNA polymerase on the cell-free protein synthesis reactions, the initial optimization process was followed by experiments concerning the supplementation of molecular crowder macromolecule PEG to cell-free protein synthesis. It was reported that PEG components can impact mRNA folding [50] and can therefore decrease the dissociation rate of T7 RNA polymerase from mRNA molecules [51]. Thus, we decided to evaluate the impact of different molecular size PEG molecules (PEG 3350, PEG 5000, PEG 20000) using two concentrations, 1% and 2%, in cell-free protein synthesis based on CHO cell lysate in the presence of 1U/μl T7 RNA polymerase (Fig 3). A reduction of T7 RNA polymerase is favorable to reduce the overall costs of the system, as supplementation costs for T7 RNA polymerase are approximately 25000 times higher compared to PEG addition. Therefore 1 U/μl T7 RNA polymerase was used during synthesis in the presence of PEG. A coupled transcription translation reaction was performed using pIX3.0-CRPV(GCT)-eYFP DNA template. The fluorescence signal of synthesized proteins was detected on μ-Ibidi slides (Fig 3A) and analyzed by Image Quant TL software quantification. The most intense fluorescence was observed adding 2% of PEG 5000, that was 2.5 and 1.8 fold higher compared to 1 U/μl and 3 U/μl T7 RNA polymerase controls, respectively (Fig 3B). A concentration dependent increase of the fluorescence signal was achieved using PEG 3350 and 5000 molecules. In the case of PEG 20000 an inhibitory effect to cell-free protein synthesis was detected for both tested concentrations. This effect was analyzed using different batches of lysate (S2 Fig). For all lysates an increase of protein yield could be detected by using PEG 3350 and PEG 5000 in comparison to 1 U/μl T7 RNA polymerase control.

### Cell-free synthesis of proteins using linear DNA templates

**Fig 4:**
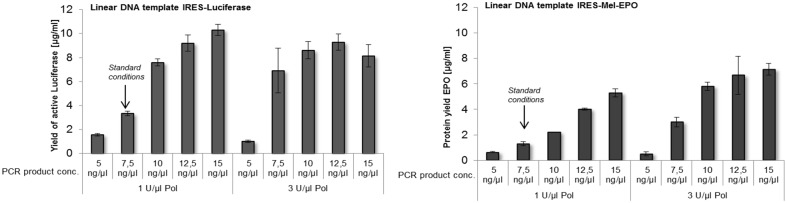
Linear DNA templates in cell-free protein synthesis based on CHO cell lysate. Linear IRES-luciferase and IRES-Mel-EPO templates tested during cell-free protein synthesis reactions in the presence of ^14^C leucine. Different concentrations of linear DNA product and T7 RNA polymerase (Pol) were added to the individual reactions. Protein yield was quantified by hot TCA precipitation followed by scintillation measurement. Error bars show standard deviations calculated from triplicates.

Besides the use of circular DNA templates, linear templates generated by PCR entail a high potential for easy and fast *in vitro* protein synthesis. During this study, linear DNA templates of luciferase and Mel-EPO, containing an CRPV-IRES and GCT as start codon, were generated by expression PCR and directly used for cell-free protein synthesis. Different concentrations of PCR product and T7 RNA polymerase were analyzed to obtain maximum protein yields. Basic conditions used for cell-free synthesis in *Sf*21 cell lysates were based on a final concentration of 7.5 ng/μl PCR product and 1 U/μl T7 RNA polymerase (Fig 4). An increase in protein yield was observed for both DNA constructs during stepwise enhancement of PCR product concentration. Increasing T7 RNA polymerase concentrations also led to a significantly improved protein synthesis (Fig 4). A plateau of protein yield could be detected by using 12.5 ng/μl and 15 ng/μl PCR product and 3 U/μl T7 RNA polymerase for both DNA templates. Maximum protein yields were obtained using 15 ng/μl PCR-product and 1 U/μl T7 RNA polymerase for linear IRES-luciferase DNA template and 15 ng/μl PCR-product and 3 U/μl T7 RNA polymerase for the linear IRES-Mel-EPO DNA template. An increase in T7 RNA polymerase concentration led to significantly higher proteins yields based on the IRES-Mel-EPO DNA template. In contrast, when using IRES-luciferase DNA template an increase of protein yield could only be obtained for PCR product concentrations of 7.5 ng/μl and 10 ng/μl. Using 12.5 ng/μl PCR product protein yield is not affected by T7 RNA polymerase concentration. Interestingly, a slight decrease of protein concentration could be detected in the sample containing 15 ng/μl PCR product upon increasing T7 RNA polymerase concentration (Fig 4).

### Synthesis of different types of protein and accumulation of proteins in microsomal structures

**Fig 5:**
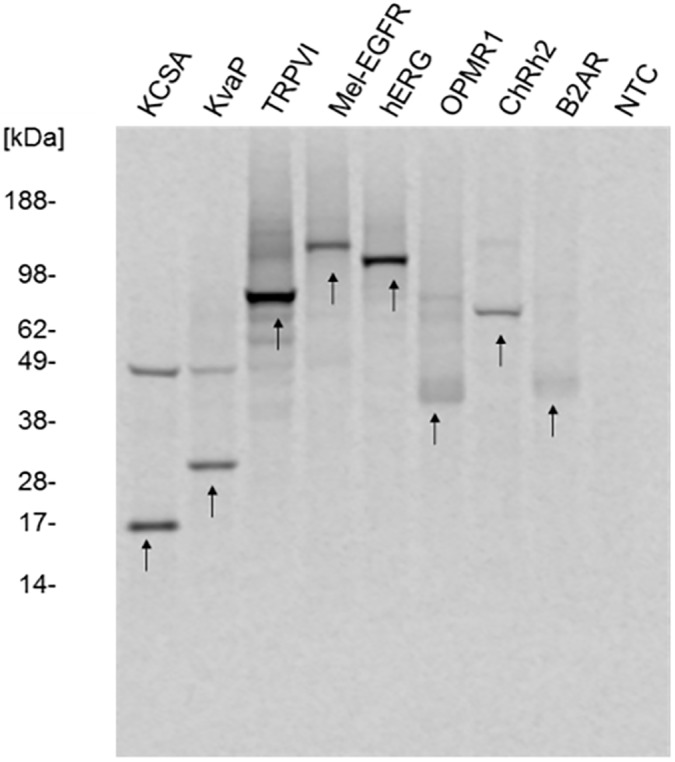
Analysis of various types of proteins synthesized in CHO cell lysates. Standard cell-free protein synthesis was performed using pIX3.0-CPRV(GCT) plasmid backbones containing the different genes of interest. ^14^C leucine labeled proteins were precipitated in acetone, separated by SDS-PAGE and visualized by autoradiography. Produced proteins are listed in Table 2. Arrows indicate the expected protein band of each individual protein. No template control (NTC) contains translation mixture without template to visualize the background translational activity of the CHO lysate.

To evaluate the performance of the CHO cell-free system, various so called “difficult-to-express” proteins were chosen for protein synthesis. Templates used in the cell-free system comprised the pH-gated potassium channel KcSA (KcsA), voltage-gated potassium uptake system KvaP (KvAP), transient receptor potential cation channel subfamily V member 1 (TRPV1), epidermal growth factor receptor (EGFR), potassium voltage-gated channel subfamily H member 2 (hERG), mu-type opioide receptor (OPMR1), channel rhodopsin-2 (ChRh2) and the beta-2 adrenergic receptor (B2AR). General characteristics of all produced proteins are illustrated in Table 2. Protein encoding DNA-fragments were integrated into pIX3.0 *in vitro* translation vector backbone harboring the CRPV-IRES at the 5´ UTR as well as the GCT start codon. For all proteins analyzed in the CHO cell-free system, the most prominent protein band of each sample corresponded to the expected molecular weight of the individual *de novo* synthesized protein (Fig 5). Analysis of KcsA, KvAP and ChRh2 revealed one additional protein band in each of the samples, indicating multimerisation of membrane proteins which was already demonstrated in the case of KcsA produced in a cell-free system based on *Sf*21 cell lysates [52]. Tetramerisation is required to get functionally active KcsA [52]. With respect to these results the second protein band that was detected in the KvAP and ChRh2 sample may give a hint of dimer formation of these proteins during the *in vitro* translation process. Initial results for functional analysis of the EGF receptor showed that proteins possess intrinsic kinase activity (S3 Fig). Autophosphorylation of EGFR at tyrosine residue could be detected by performing a kinase assay followed by immunoblotting. This result provides an initial indication for functional activity of “difficult-to-express” proteins synthesized in CHO cell-free system.

**Table 2:** “Difficult-to-express” proteins synthesized in the CHO lysate based cell-free system.

Name of protein	Gene name (Abbreviation)	Type of protein	Organism	Molecular mass [kDa]	Identifier (Uniprot)
pH-gated potassium channel KcsA	KcsA	Potassium channel	*Streptomyces lividans*	17.6	P0A334
Voltage-gated potassium uptake system KvaP	KvAP	Voltage-gated potassium channel complex	*Shewanella oneidensis*	30.8	Q8EAX3
Transient receptor potential cation channel subfamily V member 1	TRPVI	Calcium channel	*Homo sapiens*	94.9	Q8NER1
Epidermal growth factor receptor	EGFR	Transmembrane receptor protein tyrosine kinase	*Homo sapiens*	134.2	P00533
Potassium voltage-gated channel subfamily H member 2	hERG	Voltage gated Potassium channels	*Homo sapiens*	126.6	Q12809
Mu-type opioid receptor	OPMR1	G-protein coupled receptor	*Homo sapiens*	44.7	P35372
Channelrhodopsin-2	ChRh2	Ion channel	*Volvox carteri f*. *nagariensis*	77.8	B4Y105
Beta-2 adrenergic receptor	B2AR	G-protein coupled receptor	*Homo sapiens*	46.4	P07550

**Fig 6:**
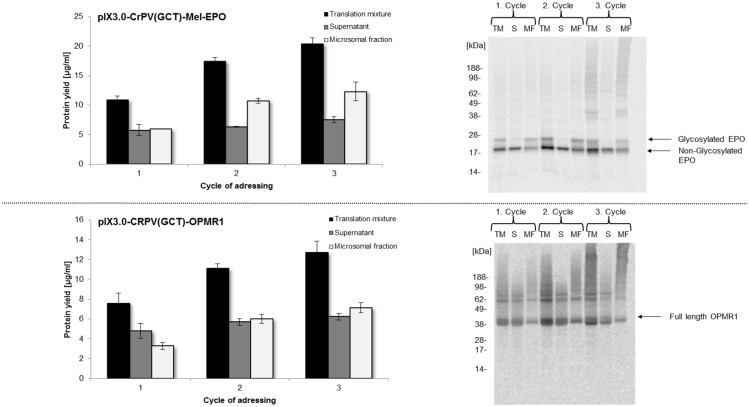
Enrichment of membrane proteins in microsomal fractions of CHO lysate using a repetitive vesicle addressing procedure. During the first synthesis step (Cycle 1) Mel-EPO and OPMR1 protein was produced according to the optimized conditions (3 U/μl T7 RNA polymerase). After completing the cell-free reaction, translation mixture was separated into microsomal fraction and supernatant by centrifugation (15 min, 4°C and 16000xg) in a standard table top centrifuge. Supernatant was removed and microsomal fraction was resuspended using freshly prepared translation mixture containing CHO cell lysate without microsomal structures to initiate the second cell-free translation cycle (Cycle 2). This procedure was repeated again, after finishing the second translation step to obtain a third step of addressing the microsomal fraction (Cycle 3). Samples of translation mixture (TM), supernatant (S) and microsomal fraction (MF) were collected after each cycle for further analysis. Protein yields were quantified by hot TCA precipitation of ^14^C leucine labeled cell-free produced proteins followed by scintillation measurement. Error bars show standard deviations calculated from triplicate analysis. Molecular weight and modifications of proteins were visualized by SDS-PAGE separation and autoradiography.

Previous studies in *Sf*21 cell lysates have shown cotranslational translocation of membrane proteins and posttranslationally modified proteins into endogenous microsomes of lysates [36]. These microsomes are generated by a mild disruption procedure during cell lysate preparation. Brödel et. al. have reported that also CHO lysates contain addressable microsomes [47]. Therefore multiple options to synthesize posttranslationally modified proteins and transmembraneous proteins are offered. Here we exemplarily demonstrate an enrichment of the glycoprotein Mel-EPO and the seven transmembrane receptor OPMR1 in microsomes of CHO cell lysates during the *in vitro* translation process. The experimental setup required a multiple addressing of the microsomal fraction by repetitive cycles of translation reaction with one batch of microsomes (Fig 6). This type of procedure was already demonstrated using the *Sf*21 cell-free protein synthesis system [22]. Detailed analysis of the translation mixture, the supernatant after centrifugation and the microsomal fraction was performed after each cycle of microsome addressing. Synthesis levels of Mel-EPO and OPMR1 in the microsomal fraction shifted from 6 μg/ml (EPO) and 3.5 μg/ml (OPMR1) after the first cycle of cell-free translation to 12.5 μg/ml (EPO) and 7.5 μg/ml (OPMR1) after the third cycle. Only a slight increase of protein yields was observed when comparing cycle 2 and cycle 3 for both proteins. These results were confirmed by autoradiography (Fig 6). As expected, target proteins showed an apparent molecular mass of 21 kDa and 44 kDa in the SDS-PAGE gel (Fig 6). As expected, *in vitro* translation of Mel-EPO resulted in an additional protein band migrating around 25 kDa, which could only be detected in the translation mixture and the vesicular fraction, but not in the supernatant fraction. Analysis of repetitive OPMR1 synthesis revealed similar results. A protein band migrating at a slightly higher apparent molecular mass than expected was observed and the intensity of this band increased during the second repetitive cycle. With respect to these observations, we were able to show the enrichment of Mel-EPO and OPMR1 in microsomal fractions of CHO cell lysates and a significant increase in glycosylated protein.

### Residue specific fluorescence labeling of proteins

**Fig 7:**
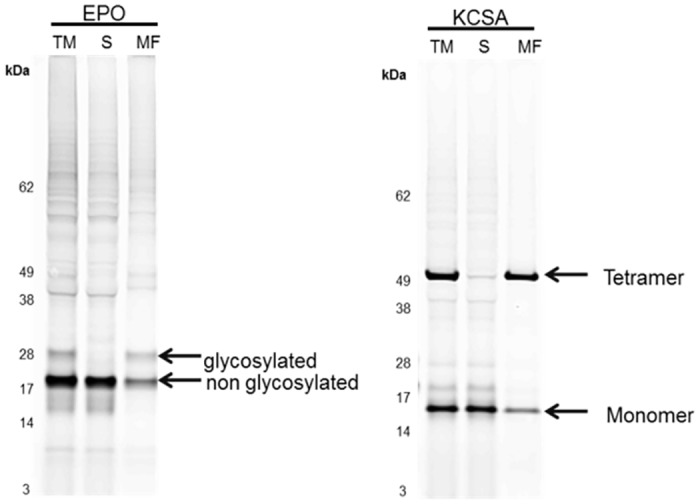
Residue specific fluorescence labeling of Mel-EPO and KCSA in cell-free systems based on translationally active CHO cell lysate. Cell-free protein synthesis based on CHO cell extracts was carried out in the presence of BODIPY-TMR-tRNA(Phe) to allow the fluorescence labeling of *de novo* synthesized Mel-EPO and KCSA. Produced glycosylated and transmembrane proteins were separated by SDS-PAGE. In-gel fluorescence was detected using a variable mode imager (Typhoon Trio Plus, GE Healthcare).

In general, one of the advantages of cell-free protein synthesis is the open character of the system. Therefore, supplementation of non-canonical amino acids harboring fluorescence molecules or special sugar moieties becomes feasible. Incorporation of non canonical amino acids into protein structure was already presented for different types of cell-free protein synthesis systems including *E*. *coli*, wheat germ and *Sf*21 lysate based reactions [53–55]. In order to analyze the incorporation of non canonical amino acids using CHO lysates we supplemented BODIPY-TMR-Lysine-tRNA(Phe) to cell-free reactions. In theory, amino acid specific integration of BODIPY-TMR-tRNA at phenylalanine codons should result in efficient labeling of the cell-free synthesized proteins due to the high sensitivity of fluorescence probes [55,56]. This is a simple, feasible and alternative procedure to commonly used ^14^C leucine labeling of synthesized proteins. In this study we have analyzed fluorescence labeling of cell-free synthesized Mel-EPO and KcSA. Labeled proteins were analyzed by separation on SDS-PAGE gels followed by in-gel fluorescence analysis (Fig 7). For both synthesis reactions bands were detected at the expected molecular mass of the proteins in each fraction (translation mixture, supernatant, microsomal fraction). Labeling of Mel-EPO revealed the expected protein pattern of additional glycosylated proteins migrating at slightly higher molecular mass in the translation mixture as well as in the microsomal fraction. The tetrameric structure of fluorescently labeled KcSA was observed in the translation mixture and in the microsomal fraction exhibiting a molecular mass of around 54 kDa.

## Discussion and Conclusion

Cell-free systems based on CHO cell lysates exhibit a tremendous potential for biotechnological applications in particular for the production of difficult-to-express proteins. The combination of well-known character and best-known safety aspects of CHO cells and the versatile cell-free technology bears many advantages for recombinant protein production.

In this study, we presented the potential of this platform with respect to different DNA templates, including various vector backbones as well as linear DNA templates, and further optimization strategies to increase the protein yield. Furthermore, we demonstrated the synthesis of different classes of proteins including ion channels, integral membrane protein receptors and G protein coupled receptors (GPCR).

Nowadays, a broad range of vector backbones are available that can be used for cell-free protein synthesis. Special requirements need to be fulfilled to enable a coupled transcription and translation reaction in cell-free systems. Commonly, transcription reactions are often conducted by using T7 RNA polymerase. Therefore the T7 promotor needs to be included in the 5´ UTR of the DNA template. Former studies revealed that cell-free systems based on CHO cell lysates showed most efficient translation rates by using an internal ribosomal entry site (IRES) in the 5´ UTR of the gene to be expressed [48]. In this study, different IRES elements were tested for their performance. In this context, it was found that IRES secondary structures of the intergenic region (IGR) of the cricket paralysis virus (CRPV) resulted in highest protein yields in the CHO cell-free system [48] which are triggered by cap independent and initiation factor independent translation initiation mechanisms [57]. By changing the start codon from ATG to GCT in the appropiate DNA template total protein yields were further increased [48]. To show the flexibility of our CHO cell-free system, we tested different commercially available vector backbones suitable for cell-free and cell-based systems. We aimed to analyze the flexibility of template acceptance in the CHO cell-free system. All templates including an IRES could be used for cell-free protein synthesis. Significant differences of protein yields were detected comparing the optimized pIX3.0-CRPV(GCT)-Luc vector to other vector backbones (pcDNA3.1 (Thermoscientific), pGEM, pT7CFE1 (Thermoscientific)). In general, CRPV IRES plasmids pIX3.0-CRPV(GCT)-Luc and pcDNA3.1-CRPV(GCT)-Luc displayed increased protein yields in comparison to the pGEM-EMCV-Luc and pT7CFE1-EMCV-Luc plasmids that contained the encephalomyocarditis virus (EMCV) IRES. EMCV mediated translation initiation is not completely translation factor independent and requires the binding of eIF4G/4A complex, IRES trans-acting factor 45 (ITAF 45) and polypyrimidine tract binding protein (PBT) [58,59]. Increased protein yields of proteins synthesized from EMCV containing plasmids, compared to CRPV IRES containing plasmids, indicate that our CHO lysates might be limited in active factors essential for EMCV dependent translation initiation. Former studies reported dependence of EMCV IRES elements on Mg^2+^ ions [60]. Thus, further optimization of salt concentrations including Mg^2+^ might consistently influence folding of the secondary structure of EMCV-IRES, enhance the stability of the initiation complex and thereby might increase protein production yields.

In this study, we additionally demonstrate the optimization of protein yields by adjusting plasmid and T7 RNA polymerase concentrations. So far, T7 RNA polymerase and pIX3.0 plasmid were applied in concentrations of 1U/μl and approximately 50 ng/μl, respectively, according to Broedel et. al. 2013. During the application of different vector backbones, it became obvious that plasmid concentrations needed to be adjusted in order to obtain the highest possible protein yields for each individual vector backbone. About 7 fold higher protein yields were obtained when using pT7CFE1-Luc and 250 ng/μl of DNA-template compared to standard conditions (50 ng/μl). Calculation of the molarity of different templates at maximum protein yields revealed a difference of optimum molarity (S1 Table). Initiation of transcription seems to be dependent on the structure of the individual DNA-template, a finding that is supported by previous studies [61]. Increasing of the T7 RNA polymerase concentration from 1 U/μl to 3 U/μl led to significantly higher protein yields using various DNA constructs (pcDNA3.1-CRPV(GCT)-Luc and pIX2.0-CRPV(GCT)-Mel-EPO). This effect was even more significant than increasing template concentration. Althought the efficiency of T7 RNA polymerase based RNA transcription was obviously improved, production of glycosylated EPO was not significantly enhanced. This observation might be due to additional limitations affecting the translocation of EPO into the microsomal structures present in the lysate as a high yield of protein was detected in the supernatant fraction. High solubility of non-translocated protein offers a high potential for further translocation optimization. Raising T7 RNA polymerase concentrations might lead to an increased RNA production rate which might as a consequence results in higher protein yields. High concentrations of T7 RNA polymerase could also induce a molecular crowding effect, which is a common feature in living cells [62]. It was shown before that T7 RNA polymerase and other macromolecules are positively affected by molecular crowding, thereby influencing biochemical kinetics by volume exclusion effects [63].

One of the commonly used molecular crowding reagents is poly ethylene glycol (PEG). The positive influence of PEG was demonstrated for *E*. *coli* and wheat germ based cell-free systems [64–67]. Our data indicate a positive molecular crowding effect in the CHO cell-free system. Upon application of PEG 3350 and PEG 5000 the fluorescence intensity of cell-free produced eYFP increased nearly 2-fold for each PEG concentration tested (1%, 2%) compare to standard conditions. Determined eYFP fluorescence intensity correlated with the total amount of *de novo* synthesized protein. Highest protein yields were obtained using 2% of PEG 5000. In contrast, PEG 20000 led to a significant decrease in protein yield. The high molecular weight PEG 20000 may affect the structure of endogenous proteins present in the lysate, thereby leading to protein precipitation [68] and subsequently to an inhibition of protein synthesis. Moreover, supplementation of PEG 3350 and PEG 5000 seems to be more efficient and also an economic way to increase protein yields in cell-free CHO systems compared to the cost-intensive increase of T7 RNA polymerase concentration from 1 U/μl to 3 U/μl.

Various automation strategies are currently in the focus of intense research activities pointing towards future applications of cell-free protein synthesis platforms. The need for novel high throughput protein synthesis strategies for the convenient preparation of “difficult-to-express” proteins, which are often targets for new pharmaceutical drugs, is obvious. Several automation strategies for cell-free systems have been published so far [55,69,70]. A cloning-free, fast and highly parallel synthesis of proteins can be achieved by using PCR products for cell-free protein synthesis. In this way, laborious cloning and vector design steps were circumvented [15]. By using so called expression PCR, all necessary regulatory sequences, including promoter, terminator and IRES sites, are added to the individual target gene sequence [71] and the derived PCR product usally can be directly used for cell-free protein synthesis. We have demonstrated the suitability of linear DNA fragments generated by PCR for cell-free protein synthesis based on CHO cell lysate. Standard conditions specified for coupled *Sf*21 cell-free system (7.5 ng/μl PCR product (unpublished results)) revealed only low protein yields using the CHO based system. A significant increase (3–4.5 fold) of protein yield was detected by increasing PCR product concentration (Fig 4). Increase of T7 RNA polymerase only showed slight improvement of protein yield for the IRES Luc construct. On the other hand the effect on IRES-Mel-EPO was remarkable. Reasons for that might be the increased production of mRNA and molecular crowding effects, as described in the section above.

The developed cell-free system based on CHO cell lysate provides a solid basis and future potential for high throughput applications. As mentioned before, so called “difficult-to-express” proteins, e.g. a broad range of membrane proteins, constitute the largest class of potential drug targets [72]. In this study, we have demonstrated the possibility to synthesize different classes of proteins in CHO cell-free system. First indications of ion channel multimerisation (KvAP and KcSA) and possible glycosylation of proteins (TRPV1, Mel-EGFR, OPMR1) were obtained. The potential to produce glycosylated EPO in CHO cell-free system was also demonstrated in former studies [47]. Such posttranslational modifications and spatial arrangements of proteins are the basis to obtain a high portion of correctly folded and functionally active proteins. Enrichment of proteins in microsomal fractions by repetitive addressing, that was shown before for insect cell lysate based cell-free systems [22], could also be confirmed for CHO cell-free system, which is a precondition for increasing the amount of active luminal, transmembraneous and membrane attached proteins.

An alternative labeling method to the generally used radio labeling of cell-free synthesized proteins was demonstrated by using residue specific incorporation of BODIPY-TMR-LYS. Incorporation of this fluorescent amino acids enables a rapid and sensitive detection of proteins [36,56]. BODIPY-TMR coupled tRNA was successfully recognized by the ribosomal machinery of CHO cell lysate allowing the subsequent translocation of EPO and KcSA into microsomes and modifications of proteins including glycosylation and multimerisation. The possibility of cotranslational incorporation of fluorescence dyes during cell-free protein synthesis is a beneficial feature of this platform and provides opportunities for future applications, based on ultrafast and highly sensitive protein staining. This method is easily high throughput applicable and an alternative method, compared to radio labeling of proteins, for fast analysis. A new prospective could be created in the area of site directed labeling in CHO cell free system that allows for the specific integration of a non canonical amino acid at one desired position in the protein.

In conclusion, cell-free systems based on translationally active CHO cell lysates represent a promising platform for the synthesis of “difficult-to-express” proteins. The technology offers the possibility to synthesize proteins in an easy and rapid manner. The flexibility of DNA template usage offers an enormous potential for fast and easy template screening technologies. The mammalian origin of the system is highly beneficial for the production of a broad range of industrial target proteins. Furthermore, the open character of the system and the opportunity of automation and high throughput protein synthesis constitute a tool for simplification of future pharmaceutical pre-screening applications.

## Supporting Information

S1 Fig Evaluation of T7 RNA polymerase concentration applied to cell-free protein synthesis. Different concentrations of T7 polymerase (0.5 U/μ, 1U/μl, 2 U/μl, 3 U/μl, 4 U/μl) were supplemented to cell-free protein synthesis reactions. Protein yields of active luciferase were determined by standard luciferase assay.
(TIF)

S2 Fig Influence of PEG addition to cell-free protein synthesis using different lots (1, 2, 3, 4) of CHO cell extract. Different PEG molecules (3350, 5000) were added in two concentrations (1%, 2%) to cell-free protein synthesis reaction using pIX3.0-CRPV(GCT)-eYFP as a template. 3 U/μl (increased concentration) and 1 U/μl T7 RNA polymerase concentrations (standard concentration) were added to cell-free synthesis used as control reactions ***A*.** Fluorescence signals of synthesized eYFP proteins were detected by fluorescence imaging on μ-Ibidi slides using the Typhoon Trio Plus Imager (GE Healthcare). ***B***. Quantification of fluorescence signals was accomplished by image analysis of μ-Ibidi slides by employing Image Quant TL Array Analysis software. Error bars show standard deviations that were calculated from triplicate analysis.
(TIF)

S3 Fig *In vitro* phosphorylation of tyrosine residue 1068 in cell-free synthesized EGF receptor. For analysis of autophosphorylation activity three samples were prepared containing an EGFR microsomal fraction, a no template control that consists of a microsomal fraction without synthesized protein and a microsomal fraction containing EGFR that is digested with calf intestinal phosphatase (CIP) after kinase buffer treatment. The CIP treated sample represents a specifity control for autophosphorylation. To allow for *in vitro* autophosphorylation of receptors embedded in the *CHO* microsomal membranes, microsomal fractions pelleted from 10 μl of the complete reaction mixture by centrifugation (15 min, 4°C, 16000xg) were collected and resuspended in 20 μl kinase buffer composed of 100 mM HEPES (pH 7.4), 1% glycerol, 0.1 mg/ml BSA, 5 mM MgCl2, 1.25 mM MnCl2, 0.1 mM NaVO3, 2 μM caspase inhibitor and 200 μM ATP. Incubation was carried out for 30 minutes at room temperature. Kinase reaction was followed by immunoblotting using the “IBlot Gel Transfer Device” (Life Technologies) according to the manufacturer’s instructions. Proteins were transferred from a 10% Bis-Tris SDS-PAGE (Life Technologies) to a PVDF membrane (Life Technologies). The membrane was blocked in TBS/T + 1% BSA for 4 hours and subsequently incubated with “Phospho-EGF Receptor (Tyr1068) (D7A5) XP^®^ Rabbit mAb 3777” primary antibody diluted 1:1000 overnight at 4°C. “Anti-rabbit IgG, HRP-linked Antibody 7074” diluted 1:2000 was used as a secondary antibody and detection was carried out using the “Amersham ECL Prime Western Blotting Detection Reagent” (GE Healthcare) and the “Typhoon Trio+ Variable Mode Imager” (GE Healthcare).
(TIF)

S1 Table General information of plasmids applied to cell-free protein synthesis. (TIF)

## References

[1] Wurm FM. Production of recombinant protein therapeutics in cultivated mammalian cells. Nat Biotech [Internet]. 2004;22(11):1393–8. 10.1038/nbt1026 15529164

[2] Lai T, Yang Y, Ng SK. Advances in Mammalian cell line development technologies for recombinant protein production. Pharmaceuticals (Basel) [Internet]. 2013;6(5):579–603. 10.3390/ph6050579 24276168 PMC3817724

[3] Kaufman RJ, Wasley LC, Spiliotes AJ, Gossels SD, Latt SA, Larsen GR, et al Coamplification and coexpression of human tissue-type plasminogen activator and murine dihydrofolate reductase sequences in Chinese hamster ovary cells. Molecular and Cellular Biology [Internet]. 1985;5(7):1750–9. 10.1128/MCB.5.7.1750 4040603 PMC367294

[4] Altamirano Claudia, Berrios Julio, Vergara Mauricio, Becerra Silvana. Advances in improving mammalian cells metabolism for recombinant protein production. Electronic Journal of Biotechnology; Vol 16, No 3 (2013) [Internet]. 2013. 10.2225/vol16-issue3-fulltext-2

[5] Kim JY, Kim Y, Lee GM. CHO cells in biotechnology for production of recombinant proteins: current state and further potential. Applied Microbiology and Biotechnology [Internet]. 2012;93(3):917–30. 10.1007/s00253-011-3758-5 22159888

[6] Walsh G. Biopharmaceutical benchmarks 2010. Nat Biotech [Internet]. 2010;28(9):917–24. 10.1038/nbt0910-917 20829826

[7] Jayapal K, Wlaschin K, Hu W, Yap M. Recombinant Protein Therapeutics from Cho Cells—20 Years and Counting. CHO Consortium: SBE Special Edition [Internet]. 2007:40–7.

[8] Rodrigues ME, Costa AR, Henriques M, Azeredo J, Oliveira R. Comparison of commercial serum-free media for CHO-K1 cell growth and monoclonal antibody production. International Journal of Pharmaceutics [Internet]. 2012;437(1–2):303–5. 10.1016/j.ijpharm.2012.08.002 22902388

[9] Matasci M, Hacker DL, Baldi L, Wurm FM. Recombinant therapeutic protein production in cultivated mammalian cells: current status and future prospects. Protein therapeutics [Internet]. 2008;5(2–3):e37–e42. 10.1016/j.ddtec.2008.12.003 24981089

[10] Durocher Y, Butler M. Expression systems for therapeutic glycoprotein production. Chemical biotechnology ● Pharmaceutical biotechnology [Internet]. 2009;20(6):700–7. 10.1016/j.copbio.2009.10.008 19889531

[11] Wiberg FC, Rasmussen SK, Frandsen TP, Rasmussen LK, Tengbjerg K, Coljee VW, et al Production of target-specific recombinant human polyclonal antibodies in mammalian cells. Biotechnol. Bioeng. [Internet]. 2006;94(2):396–405. 10.1002/bit.20865 16596663

[12] Klammt C, Schwarz D, Löhr F, Schneider B, Dötsch V, Bernhard F. Cell-free expression as an emerging technique for the large scale production of integral membrane protein. FEBS Journal [Internet]. 2006;273(18):4141–53. 10.1111/j.1742-4658.2006.05432.x 16930130

[13] Bechlars S, Jäckel C, Diescher S, Wüstenhagen DA, Kubick S, Dieckmann R, et al Characterization of trh2 Harbouring Vibrio parahaemolyticus Strains Isolated in Germany. PLoS ONE [Internet]. 2015;10(3):e0118559 10.1371/journal.pone.0118559 25799574 PMC4370738

[14] Bechlars S, Wüstenhagen DA, Drägert K, Dieckmann R, Strauch E, Kubick S. Cell-free synthesis of functional thermostable direct hemolysins of Vibrio parahaemolyticus. Toxicon [Internet]. 2013;76:132–42. 10.1016/j.toxicon.2013.09.012 24060377

[15] Hodgman CE, Jewett MC. Cell-free synthetic biology: Thinking outside the cell. Synthetic Biology: New Methodologies and Applications for Metabolic Engineering [Internet]. 2012;14(3):261–9. 10.1016/j.ymben.2011.09.002 PMC3322310 21946161

[16] Kim D, Kigawa T, Choi C, Yokoyama S. A Highly Efficient Cell-Free Protein Synthesis System from Escherichia coli. European Journal of Biochemistry [Internet]. 1996;239(3):881–6. 10.1111/j.1432-1033.1996.0881u.x 8774739

[17] Kim D, Swartz JR. Efficient production of a bioactive, multiple disulfide-bonded protein using modified extracts of Escherichia coli. Biotechnol. Bioeng. [Internet]. 2004;85(2):122–9. 10.1002/bit.10865 14704994

[18] Spirin A, Baranov V, Ryabova La, Ovodov S, Alakhov Y. A continuous cell-free translation system capable of producing polypeptides in high yield. Science [Internet]. 1988;242(4882):1162–4. 10.1126/science.3055301 3055301

[19] Nirenberg MW, Matthaei JH. The dependence of cell- free protein synthesis in e. Coli upon naturally occurring or synthetic polyribonucleotides. Proceedings of the National Academy of Sciences of the United States of America [Internet]. 1961;47(10):1588–602. 10.1073/pnas.47.10.1588 14479932 PMC223178

[20] Carlson ED, Gan R, Hodgman CE, Jewett MC. Cell-Free Protein Synthesis: Applications Come of Age. Biotechnology advances [Internet]. 2011;30(5):1185–94. 10.1016/j.biotechadv.2011.09.016 22008973 PMC4038126

[21] Zemella A, Thoring L, Hoffmeister C, Kubick S. Cell-Free Protein Synthesis: Pros and Cons of Prokaryotic and Eukaryotic Systems. ChemBioChem [Internet]. 2015;16(17):2420–31. 10.1002/cbic.201500340 26478227 PMC4676933

[22] Stech M, Brödel AK, Quast RB, Sachse R, Kubick S. Cell-Free Systems: Functional Modules for Synthetic and Chemical Biology In: Zeng A, editor. Fundamentals and Application of New Bioproduction Systems. Berlin, Heidelberg: Springer Berlin Heidelberg; 2013 p. 67–102. 10.1007/10_2013_185 23576054

[23] Morita EH, Sawasaki T, Tanaka R, Endo Y, Kohno T. A wheat germ cell-free system is a novel way to screen protein folding and function. Protein Science [Internet]. 2003;12(6):1216–21. 10.1110/ps.0241203 12761392 PMC2323893

[24] Madono M, Sawasaki T, Morishita R, Endo Y. Wheat germ cell-free protein production system for post-genomic research. Advances in Cell-free Protein Expression [Internet]. 2011;28(3):211–7. 10.1016/j.nbt.2010.08.009 20800705

[25] Takai K, Sawasaki T, Endo Y. Practical cell-free protein synthesis system using purified wheat embryos. Nat. Protocols [Internet]. 2010;5(2):227–38. 10.1038/nprot.2009.207 20134421

[26] Sawasaki T, Hasegawa Y, Tsuchimochi M, Kamura N, Ogasawara T, Kuroita T, et al A bilayer cell-free protein synthesis system for high-throughput screening of gene products. FEBS Letters [Internet]. 2002;514(1):102–5. 10.1016/S0014-5793(02)02329-3 11904190

[27] Tse TP, Taylor JM. Translation of albumin messenger RNA in a cell-free protein-synthesizing system derived from wheat germ. Journal of Biological Chemistry [Internet]. 1977;252(4):1272–8. 14149

[28] Hunter AR, Farrell PJ, Jackson RJ, Hunt T. The Role of Polyamines in Cell-Free Protein Synthesis in the Wheat-Germ System. European Journal of Biochemistry [Internet]. 1977;75(1):149–57. 10.1111/j.1432-1033.1977.tb11512.x 862615

[29] Pelham HRB, Jackson RJ. An Efficient mRNA-Dependent Translation System from Reticulocyte Lysates. European Journal of Biochemistry [Internet]. 1976;67(1):247–56. 10.1111/j.1432-1033.1976.tb10656.x 823012

[30] Schweet R, Lamfrom H, Allen E. The synthesis of hemoglobin in a cell-free system. Proceedings of the National Academy of Sciences of the United States of America [Internet]. 1958;44(10):1029–35. 10.1073/pnas.44.10.1029 16590302 PMC528688

[31] Browne GJ, Proud CG. Regulation of peptide-chain elongation in mammalian cells. European Journal of Biochemistry [Internet]. 2002;269(22):5360–8. 10.1046/j.1432-1033.2002.03290.x 12423334

[32] Merrick WC. Cap-dependent and cap-independent translation in eukaryotic systems. Gene [Internet]. 2004;332:1–11. 10.1016/j.gene.2004.02.051 15145049

[33] John W. B. Hershey, William C. Merrick. 2 The Pathway and Mechanism of Initiation of Protein Synthesis. Cold Spring Harbor Monograph Archive; Volume 39 (2000): Translational Control of Gene Expression. 2000. Available: https://cshmonographs.org/index.php/monographs/article/view/3233. 11097426

[34] MacDonald MR, McCourt DW, Krause JE. Posttranslational processing of alpha-, beta-, and gamma-preprotachykinins. Cell-free translation and early posttranslational processing events. Journal of Biological Chemistry [Internet]. 1988;263(29):15176–83. 3049602

[35] Harbers M. Wheat germ systems for cell-free protein expression. FEBS Letters [Internet]. 2014;588(17):2762–73. 10.1016/j.febslet.2014.05.061 24931374

[36] Sachse R, Wüstenhagen D, Šamalíková M, Gerrits M, Bier FF, Kubick S. Synthesis of membrane proteins in eukaryotic cell-free systems. Eng. Life Sci. 2013;13(1):39–48. Available: 10.1002/elsc.201100235. 10.1002/elsc.201100235

[37] Stech M, Merk H, Schenk JA, Stöcklein WF, Wüstenhagen DA, Micheel B, et al Production of functional antibody fragments in a vesicle-based eukaryotic cell-free translation system. Journal of Biotechnology [Internet]. 2013;164(2):220–31. 10.1016/j.jbiotec.2012.08.020 22982167

[38] Quast RB, Claussnitzer I, Merk H, Kubick S, Gerrits M. Synthesis and site-directed fluorescence labeling of azido proteins using eukaryotic cell-free orthogonal translation systems. Analytical Biochemistry. 2014;451(0):4–9. Available: http://www.sciencedirect.com/science/article/pii/S0003269714000335. 10.1016/j.ab.2014.01.013 24491444

[39] Kubick S, Schacherl J, Fleischer-Notter H, Royall E, Roberts LO, Stiege W. In Vitro Translation in an Insect-Based Cell-Free System In: Swartz JR, editor. Cell-Free Protein Expression. Berlin, Heidelberg: Springer Berlin Heidelberg; 2003 p. 209–17. 10.1007/978-3-642-59337-6_25

[40] Fenz SF, Sachse R, Schmidt T, Kubick S. Cell-free synthesis of membrane proteins: Tailored cell models out of microsomes. Biochimica et Biophysica Acta (BBA)—Biomembranes. 2014;1838(5):1382–8. Available: http://www.sciencedirect.com/science/article/pii/S0005273613004483. 10.1016/j.bbamem.2013.12.009 24370776

[41] Komoda K, Naito S, Ishikawa M. Replication of plant RNA virus genomes in a cell-free extract of evacuolated plant protoplasts. Proceedings of the National Academy of Sciences of the United States of America. 2003;101(7):1863–7. Available: http://www.ncbi.nlm.nih.gov/pmc/articles/PMC357018/. 10.1073/pnas.0307131101 PMC357018 14769932

[42] Gursinsky T, Schulz B, Behrens S. Replication of Tomato bushy stunt virus RNA in a plant in vitro system. Virology. 2009;390(2):250–60. Available: http://www.sciencedirect.com/science/article/pii/S0042682209003110. 10.1016/j.virol.2009.05.009 19520410

[43] Buntru M, Vogel S, Stoff K, Spiegel H, Schillberg S. A versatile coupled cell-free transcription–translation system based on tobacco BY-2 cell lysates. Biotechnol. Bioeng. [Internet]. 2015;112(5):867–78. 10.1002/bit.25502 25421615

[44] Buntru M, Vogel S, Spiegel H, Schillberg S. Tobacco BY-2 cell-free lysate: an alternative and highly-productive plant-based in vitro translation system. BMC Biotechnology [Internet]. 2014;14(1):1–11. 10.1186/1472-6750-14-37 24886601 PMC4101825

[45] Kobayashi T, Machida K, Imataka H. Human Cell Extract-Derived Cell-Free Systems for Virus Synthesis In: Alexandrov K, Johnston WA, editors. Cell-Free Protein Synthesis. Methods in Molecular Biology: Humana Press; 2014 p. 149–56. 10.1007/978-1-62703-782-2_9 24395414

[46] Brödel AK, Kubick S. Developing cell-free protein synthesis systems: a focus on mammalian cells. Pharmaceutical Bioprocessing [Internet]. 2014;2(4):339–48. 10.4155/pbp.14.30

[47] Brödel AK, Sonnabend A, Kubick S. Cell-free protein expression based on extracts from CHO cells. Biotechnol. Bioeng. [Internet]. 2014;111(1):25–36. 10.1002/bit.25013 24018795

[48] Brödel AK, Sonnabend A, Roberts LO, Stech M, Wüstenhagen DA, Kubick S. IRES-Mediated Translation of Membrane Proteins and Glycoproteins in Eukaryotic Cell-Free Systems. PLoS ONE [Internet]. 2013;8(12):e82234 10.1371/journal.pone.0082234 24376523 PMC3869664

[49] Orth JH, Schorch B, Boundy S, Ffrench-Constant R, Kubick S, Aktories K. Cell-free synthesis and characterization of a novel cytotoxic pierisin-like protein from the cabbage butterfly Pieris rapae. Toxicon [Internet]. 2011;57(2):199–207. 10.1016/j.toxicon.2010.11.011 21112350

[50] Strulson CA, Boyer JA, Whitman EE, Bevilacqua PC. Molecular crowders and cosolutes promote folding cooperativity of RNA under physiological ionic conditions. RNA [Internet]. 2014;20(3):331–47. 10.1261/rna.042747.113 24442612 PMC3923128

[51] Tabaka M, Kalwarczyk T, Hołyst R. Quantitative influence of macromolecular crowding on gene regulation kinetics. Nucleic Acids Research [Internet]. 2013;42(2):727–38. 10.1093/nar/gkt907 24121687 PMC3902910

[52] Dondapati SK, Kreir M, Quast RB, Wüstenhagen DA, Brüggemann A, Fertig N, et al Membrane assembly of the functional KcsA potassium channel in a vesicle-based eukaryotic cell-free translation system. Biosensors and Bioelectronics. 2014;59(0):174–83. Available: http://www.sciencedirect.com/science/article/pii/S0956566314001730. 10.1016/j.bios.2014.03.004 24727603

[53] Hong SH, Kwon Y, Jewett MC. Non-standard amino acid incorporation into proteins using Escherichia coli cell-free protein synthesis. Frontiers in Chemistry [Internet]. 2014;2 10.3389/fchem.2014.00034 PMC4050362 24959531

[54] Abe M, Ohno S, Yokogawa T, Nakanishi T, Arisaka F, Hosoya T, et al Detection of structural changes in a cofactor binding protein by using a wheat germ cell-free protein synthesis system coupled with unnatural amino acid probing. Proteins: Structure, Function and Genetics [Internet]. 2007;67(3):643–52. 10.1002/prot.21341 17348022

[55] Quast RB, Mrusek D, Hoffmeister C, Sonnabend A, Kubick S. Cotranslational incorporation of non-standard amino acids using cell-free protein synthesis. FEBS Letters [Internet]. 2015;589(15):1703–12. 10.1016/j.febslet.2015.04.041 25937125

[56] Gerrits M, Merk H, Stiege W, Erdmann VA. Towards Improved Applications of Cell-Free Protein Biosynthesis—The Influence of mRNA Structure and Suppressor tRNAS on the Efficiency of the System In: Barciszewski J, Clark BFC, editors. RNA Biochemistry and Biotechnology. Dordrecht: Springer Netherlands; 1999 p. 335–45. 10.1007/978-94-011-4485-8_25

[57] Jan E. Divergent IRES elements in invertebrates. Translational Control During Virus Infections [Internet]. 2006;119(1):16–28. 10.1016/j.virusres.2005.10.011 16307820

[58] Pestova TV, Hellen CU, Shatsky IN. Canonical eukaryotic initiation factors determine initiation of translation by internal ribosomal entry. Molecular and Cellular Biology [Internet]. 1996;16(12):6859–69. 10.1128/MCB.16.12.6859 8943341 PMC231689

[59] Hellen CU, Sarnow P. Internal ribosome entry sites in eukaryotic mRNA molecules. Genes & Development [Internet]. 2001;15(13):1593–612. 10.1101/gad.891101 11445534

[60] Dupont JA, Snoussi K. Mg(2+) modulation of EMCV IRES key activity fragment equilibria and r(G·C) base-pair kinetics. Journal of Biological Physics [Internet]. 2009;35(3):231–43. 10.1007/s10867-009-9151-2 19669575 PMC2710458

[61] Schenborn ET, Mierendorf RC. A novel transcription property of SP6 and T7 RNA polymerases: dependence on template structure. Nucleic Acids Research [Internet]. 1985;13(17):6223–36. 10.1093/nar/13.17.6223 2995921 PMC321948

[62] Fujimoto T, Nakano S, Miyoshi D, Sugimoto N. The Effects of Molecular Crowding on the Structure and Stability of G-Quadruplexes with an Abasic Site. Journal of Nucleic Acids [Internet]. 2011;2011:857149 10.4061/2011/857149 21949901 PMC3178115

[63] Tan C, Saurabh S, Bruchez MP, Schwartz R, Leduc P. Molecular crowding shapes gene expression in synthetic cellular nanosystems. Nat Nano [Internet]. 2013;8(8):602–8. 10.1038/nnano.2013.132 23851358 PMC3951305

[64] Nakano H, Tanaka T, Kawarasaki Y, Yamane T. Highly productive cell-free protein synthesis system using condensed wheat-germ extract. Journal of Biotechnology [Internet]. 1996;46(3):275–82. 10.1016/0168-1656(96)00022-3

[65] Nakano H, Tanaka T, Kawarasaki Y, Yamane T. An increased rate of cell-free protein synthesis by condensing wheat-germ extract with ultrafiltration membranes. Bioscience, Biotechnology and Biochemistry [Internet]. 1994;58(4):631–4. 10.1080/bbb.58.631 7764855

[66] Kigawa T, Yabuki T, Matsuda N, Matsuda T, Nakajima R, Tanaka A, et al Preparation of Escherichia coli cell extract for highly productive cell-free protein expression. Journal of Structural and Functional Genomics [Internet]. 2004;5(1):63–8. 10.1023/B:JSFG.0000029204.57846.7d 15263844

[67] Kigawa T, Yabuki T, Yoshida Y, Tsutsui M, Ito Y, Shibata T, et al Cell-free production and stable-isotope labeling of milligram quantities of proteins. FEBS Letters [Internet]. 1999;442(1):15–9. 10.1016/S0014-5793(98)01620-2 9923595

[68] Atha DH, Ingham KC. Mechanism of precipitation of proteins by polyethylene glycols. Analysis in terms of excluded volume. Journal of Biological Chemistry [Internet]. 1981;256(23):12108–17. 7298647

[69] Makino S, Beebe ET, Markley JL, Fox BG. Cell-Free Protein Synthesis for Functional and Structural Studies In: Chen WY, editor. Structural Genomics: General Applications. Totowa, NJ: Humana Press; 2014 p. 161–78. 10.1007/978-1-62703-691-7_11 24203331

[70] Aoki M, Matsuda T, Tomo Y, Miyata Y, Inoue M, Kigawa T, et al Automated system for high-throughput protein production using the dialysis cell-free method. Protein Expression and Purification [Internet]. 2009;68(2):128–36. 10.1016/j.pep.2009.07.017 19664715

[71] Merk H, Meschkat D, Stiege W. Expression-PCR: from Gene Pools to Purified Proteins Within 1 Day In: Swartz JR, editor. Cell-Free Protein Expression. Berlin, Heidelberg: Springer Berlin Heidelberg; 2003 p. 15–23. 10.1007/978-3-642-59337-6_3

[72] Arinaminpathy Y, Khurana E, Engelman DM, Gerstein MB. Computational analysis of membrane proteins: the largest class of drug targets. Drug Discovery Today [Internet]. 2009;14(23–24):1130–5. 10.1016/j.drudis.2009.08.006 19733256 PMC2796609

