# Supplementary figures and images for: Cell-Free Systems Based on CHO Cell Lysates: Optimization Strategies, Synthesis of “Difficult-to-Express” Proteins and Future Perspectives
Source: PLoS One. 2016 Sep 29;11(9):e0163670. doi: 10.1371/journal.pone.0163670 (PMC5042383; doi:10.1371/journal.pone.0163670)

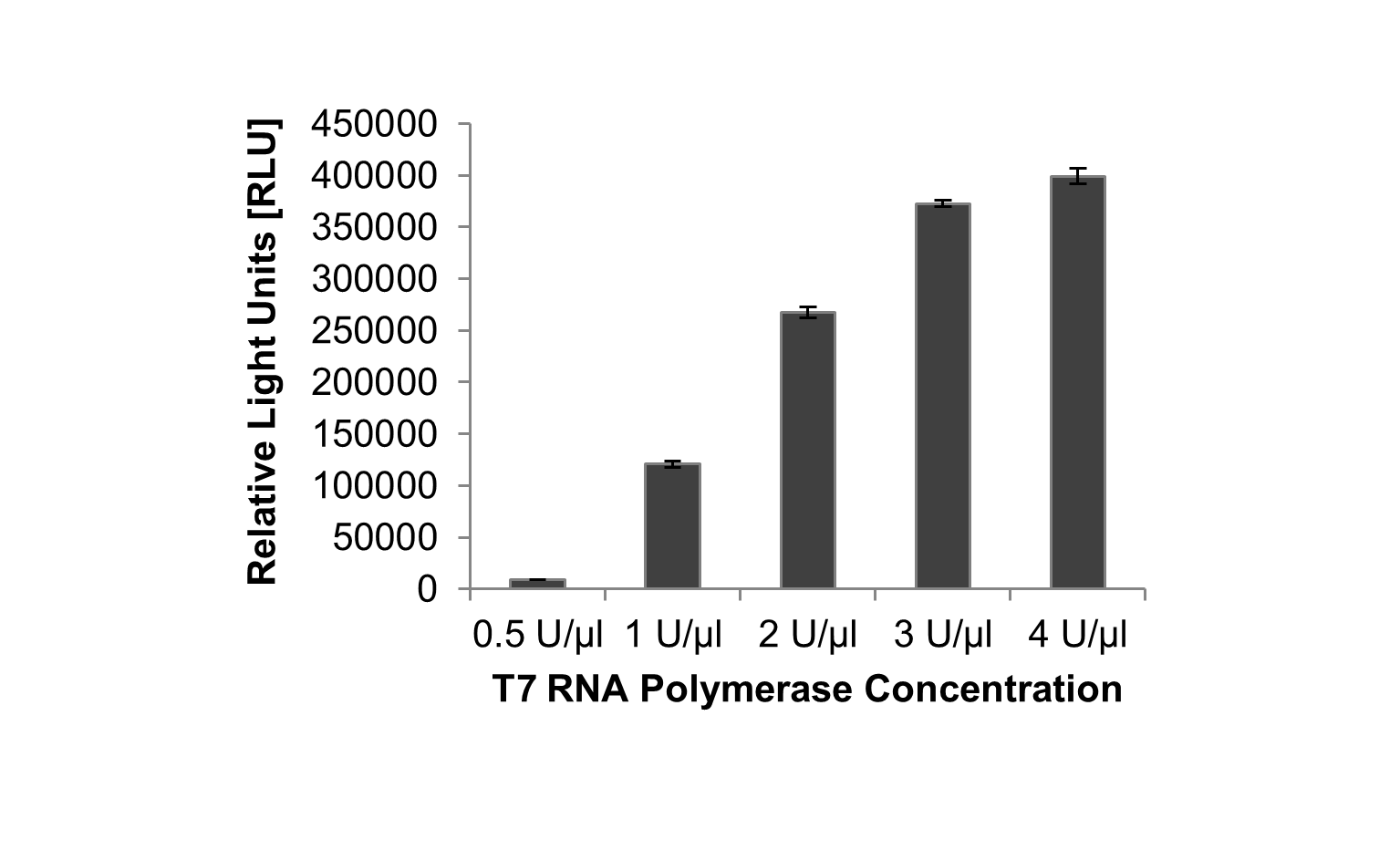

Supplement: S1 Fig — Different concentrations of T7 polymerase (0.5 U/μ, 1U/μl, 2 U/μl, 3 U/μl, 4 U/μl) were supplemented to cell-free protein synthesis reactions. Protein yields of active luciferase were determined by standard luciferase assay. (TIF) [file pone.0163670.s001.tif]

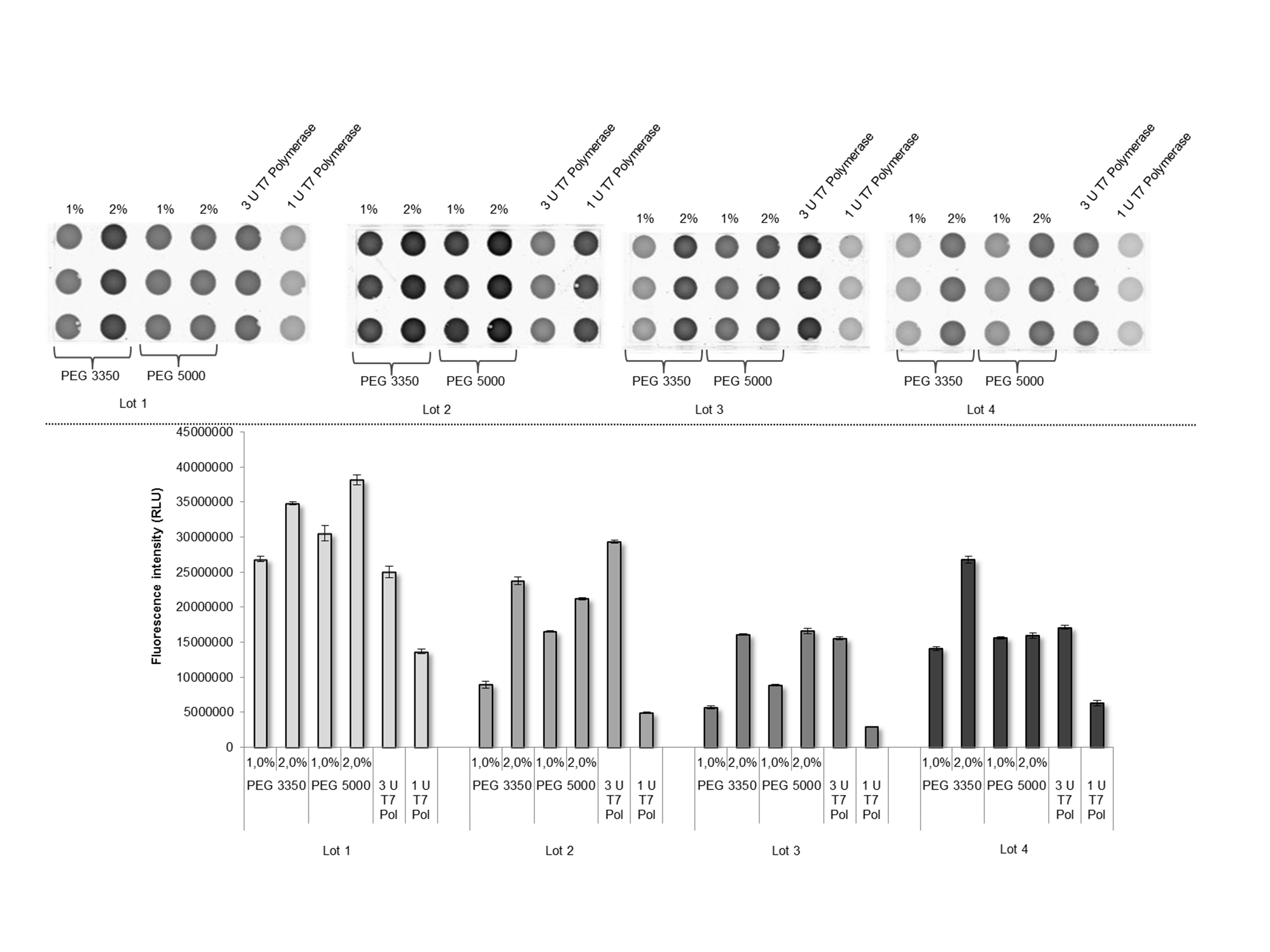

Supplement: S2 Fig — Different PEG molecules (3350, 5000) were added in two concentrations (1%, 2%) to cell-free protein synthesis reaction using pIX3.0-CRPV(GCT)-eYFP as a template. 3 U/μl (increased concentration) and 1 U/μl T7 RNA polymerase concentrations (standard concentration) were added to cell-free synthesis used as control reactions A. Fluorescence signals of synthesized eYFP proteins were detected by fluorescence imaging on μ-Ibidi slides using the Typhoon Trio Plus Imager (GE Healthcare). B. Quantification of fluorescence signals was accomplished by image analysis of μ-Ibidi slides by employing Image Quant TL Array Analysis software. Error bars show standard deviations that were calculated from triplicate analysis. (TIF) [file pone.0163670.s002.tif]

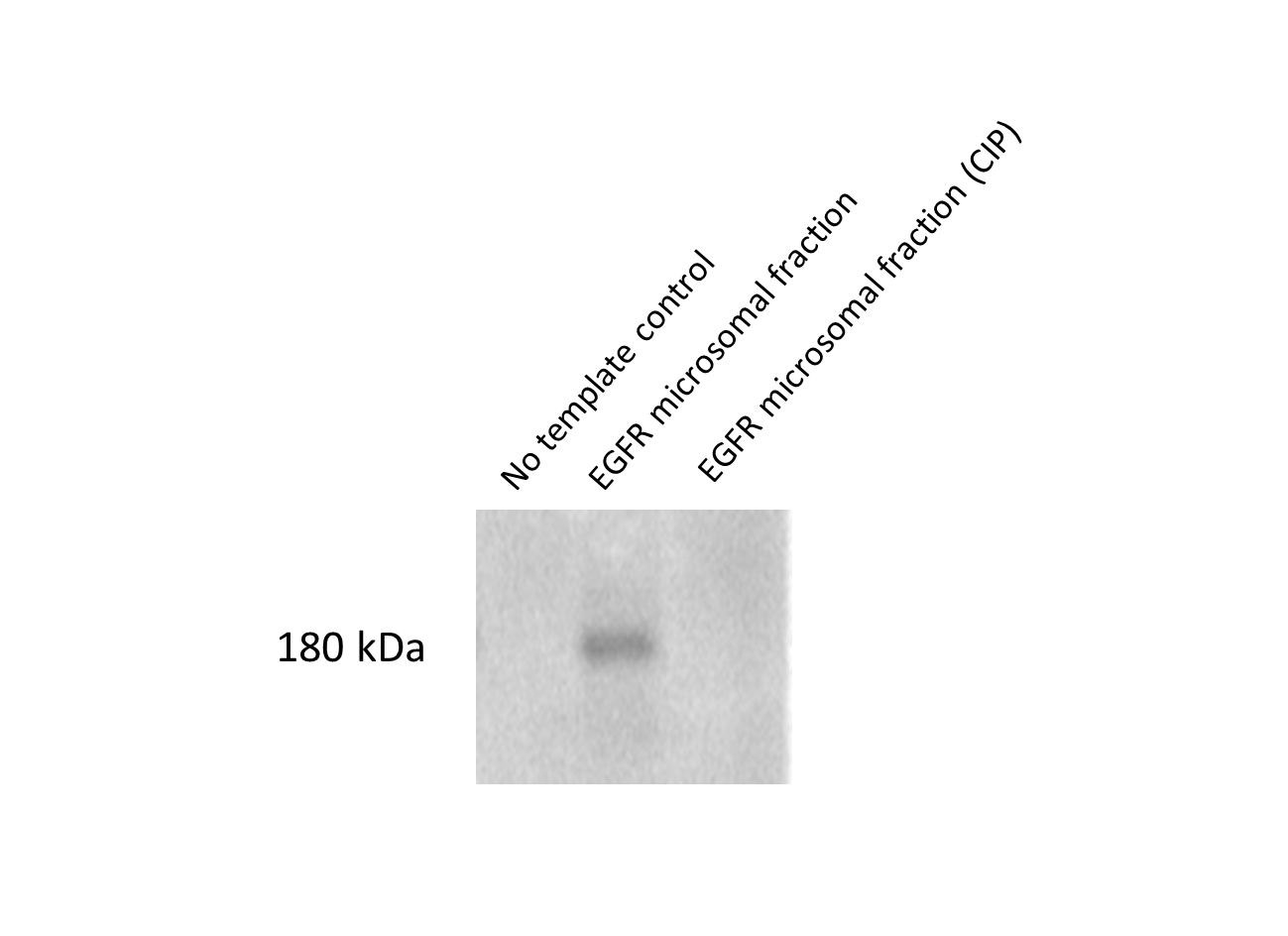

Supplement: S3 Fig — For analysis of autophosphorylation activity three samples were prepared containing an EGFR microsomal fraction, a no template control that consists of a microsomal fraction without synthesized protein and a microsomal fraction containing EGFR that is digested with calf intestinal phosphatase (CIP) after kinase buffer treatment. The CIP treated sample represents a specifity control for autophosphorylation. To allow for in vitro autophosphorylation of receptors embedded in the CHO microsomal membranes, microsomal fractions pelleted from 10 μl of the complete reaction mixture by centrifugation (15 min, 4°C, 16000xg) were collected and resuspended in 20 μl kinase buffer composed of 100 mM HEPES (pH 7.4), 1% glycerol, 0.1 mg/ml BSA, 5 mM MgCl2, 1.25 mM MnCl2, 0.1 mM NaVO3, 2 μM caspase inhibitor and 200 μM ATP. Incubation was carried out for 30 minutes at room temperature. Kinase reaction was followed by immunoblotting using the “IBlot Gel Transfer Device” (Life Technologies) according to the manufacturer’s instructions. Proteins were transferred from a 10% Bis-Tris SDS-PAGE (Life Technologies) to a PVDF membrane (Life Technologies). The membrane was blocked in TBS/T + 1% BSA for 4 hours and subsequently incubated with “Phospho-EGF Receptor (Tyr1068) (D7A5) XP® Rabbit mAb 3777” primary antibody diluted 1:1000 overnight at 4°C. “Anti-rabbit IgG, HRP-linked Antibody 7074” diluted 1:2000 was used as a secondary antibody and detection was carried out using the “Amersham ECL Prime Western Blotting Detection Reagent” (GE Healthcare) and the “Typhoon Trio+ Variable Mode Imager” (GE Healthcare). (TIF) [file pone.0163670.s003.tif]

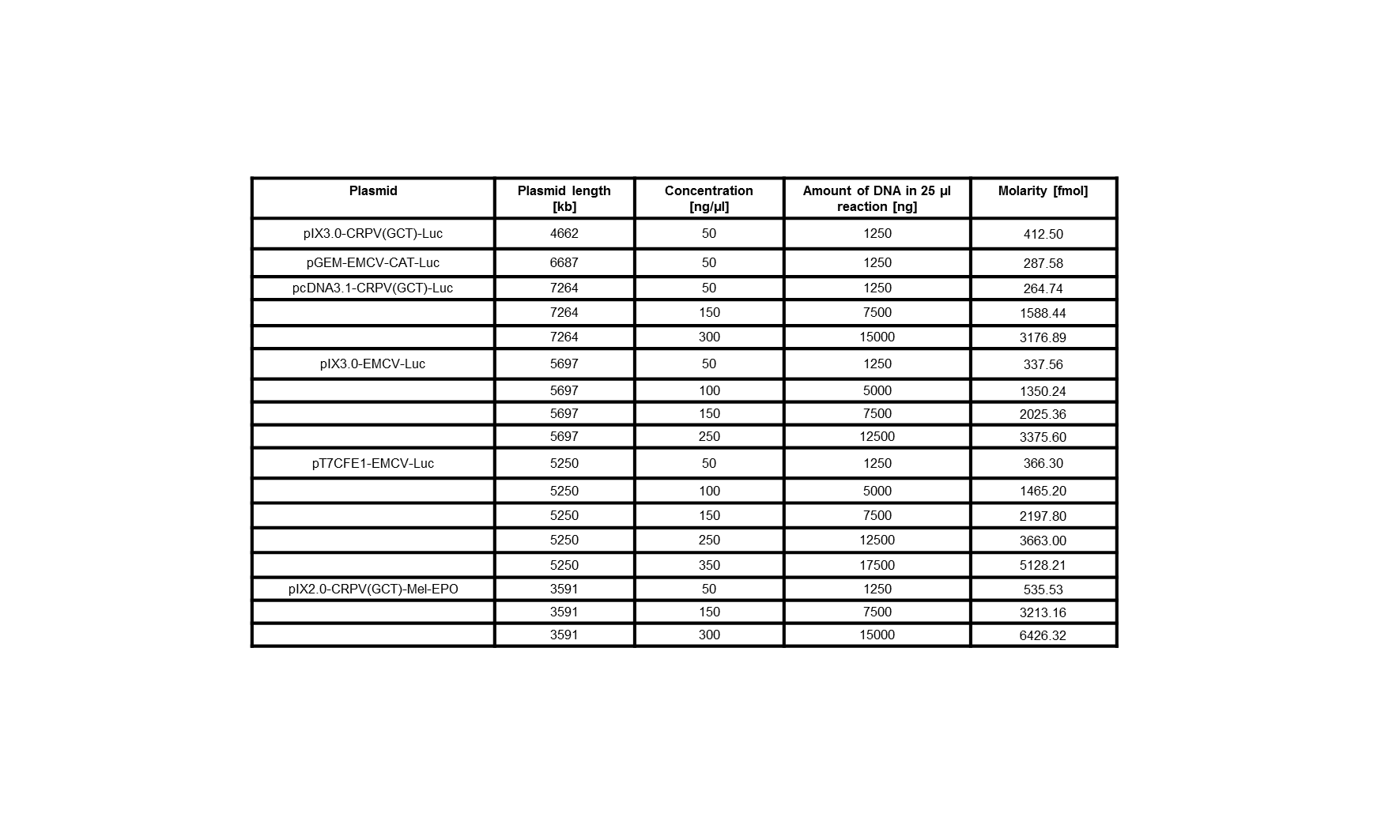

Supplement: S1 Table — (TIF) [file pone.0163670.s004.tif]
